# An Effective Skin Cancer Classification Mechanism via Medical Vision Transformer

**DOI:** 10.3390/s22114008

**Published:** 2022-05-25

**Authors:** Suliman Aladhadh, Majed Alsanea, Mohammed Aloraini, Taimoor Khan, Shabana Habib, Muhammad Islam

**Affiliations:** 1 Department of Information Technology, College of Computer, Qassim University, Buraydah 52571, Saudi Arabia; s.aladhadh@qu.edu.sa; 2 Computing Department, Arabeast Colleges, Riyadh 13544, Saudi Arabia; malsanea@arabeast.edu.sa; 3 Department of Electrical Engineering, College of Engineering, Qassim University, Unaizah 56452, Saudi Arabia; mo.aloraini@qu.edu.sa; 4 Department of Computer Science, Islamia College Peshawar, Peshawar 25120, Pakistan; taimooricp@gmail.com; 5 Department of Electrical Engineering, College of Engineering and Information Technology, Onaizah Colleges, Unaizah 56447, Saudi Arabia; m.islam@oc.edu.sa

**Keywords:** medical images, skin cancer, Medical Vision Transformer, artificial intelligence

## Abstract

Skin Cancer (SC) is considered the deadliest disease in the world, killing thousands of people every year. Early SC detection can increase the survival rate for patients up to 70%, hence it is highly recommended that regular head-to-toe skin examinations are conducted to determine whether there are any signs or symptoms of SC. The use of Machine Learning (ML)-based methods is having a significant impact on the classification and detection of SC diseases. However, there are certain challenges associated with the accurate classification of these diseases such as a lower detection accuracy, poor generalization of the models, and an insufficient amount of labeled data for training. To address these challenges, in this work we developed a two-tier framework for the accurate classification of SC. During the first stage of the framework, we applied different methods for data augmentation to increase the number of image samples for effective training. As part of the second tier of the framework, taking into consideration the promising performance of the Medical Vision Transformer (MVT) in the analysis of medical images, we developed an MVT-based classification model for SC. This MVT splits the input image into image patches and then feeds these patches to the transformer in a sequence structure, like word embedding. Finally, Multi-Layer Perceptron (MLP) is used to classify the input image into the corresponding class. Based on the experimental results achieved on the Human Against Machine (HAM10000) datasets, we concluded that the proposed MVT-based model achieves better results than current state-of-the-art techniques for SC classification.

## 1. Introduction

Over the last few years there have been substantial increases in the number of skin cancers (SC) caused by several factors such as smoking, drinking alcohol, and most importantly, the harmful UV rays emitted by the sun [1]. More than two people die of this deadly disease every hour in the United States alone. Cancer is a disease that is also deadly in other parts of the world besides the United States. Cancer occurs when cells in a part of the body grow uncontrollably, causing them to spread to other parts of the body. There are several types of cancer found in various parts of the body. SC is one of the most prevalent types of cancer and some of its variants can be potentially hazardous to human life [2]. One of the most commonly occurring types of SC is non-melanoma, while the other is melanoma. Non-melanoma cancer can usually be cured with surgery and other regularly prescribed medications, and it is usually not fatal. On the other hand, melanoma is among the most threatening types of SC since it does not have a high survival rate; however, it represents less than 5% of all SC. Globally, according to a report from the World Health Organization (WHO), there are an estimated 132,000 cases of melanoma each year. There were a total of 60,000 deaths reported in 2015 due to syphilis [3]. Considering the higher riskiness of the disease and to avoid any serious threat to human life, it is imperative that it is detected and classified in a timely manner. Self-examination and clinical examination are two basic methods of detecting the disease, and the results of the two are comparable. In a self-examination, the patient, or other person, subjectively identifies a lesion and, in most cases due to the lack of sufficient knowledge, either overreacts or underreacts. Clinical examination, on the other hand, is more expensive and requires medical experts as well as specialized medical diagnostic tools, such as dedicated laser-based devices, micro-spectroscopy, and other dermoscopy tools to locate the lesion. These tools are often very expensive and require highly skilled staff to operate them. Over the decades, researchers around the world have developed various Computer-Assisted Diagnosis (CAD) systems to assist medical experts in clinical examination as a second opinion for early SC detection [4,5]. Older CAD systems are usually based on Conventional Computer Vision-based methods (CCV), whereas modern systems use Machine/Deep Learning (DL) based techniques. The CCV-based methods mostly use handcrafted feature extraction mechanisms to extract various features, i.e., texture, color, size, and shape, to detect SC [6]. For example, Ansari et al. [7] presented a skin cancer detection system using a Support Vector Machine (SVM), which helps early detection of skin cancer disease. They used traditional image processing and featured engineering methods for effective feature selection and the Support Vector Machine (SVM) algorithm to classify them.

A study conducted by TaufiSq et al. [8] described a real-time application for detecting skin cancer using a smartphone. Several texture features were extracted, such as area, perimeter, and eccentricity, and then fed these texture features into an SVM for classification. The next step was completed by Alfed et al. [9] by extracting textural and color features for the classification of skin lesions. As a result, they concluded that a Histogram of Gradients (HOG) and a Histogram of Lines (HL) are better suited for the analysis and classification of dermoscopic images. The rules used by Alquran et al. [10] to extract features were Asymmetry, Border, Color, and Diameter (ABCD), and the principal component analysis (PCA) method was used to select the most prominent features from these. Finally, an SVM classifier was used to determine whether a lesion was malignant or benign. As per Thanh et al. [11] proposed three stages for skin lesion analysis: pre-processing by relying on adaptive principal curvature, segmenting skin lesions by means of color normalization, and extracting features by the ABCD rule. According to Victor et al. [12], there are four main steps in the classification of SC including (1) pre-processing (2) segmentation (3) feature extraction and (4) classification. Performance evaluation was carried out using four different classifiers, such as Decision Tree (DT), K-Nearest Neighbor (KNN), Boosted Decision Tree (BT) and SVM. Typically, these techniques were used before 2010 and between 2010 and 2017 [13]. Javed et al. [14], proposed a statistical histogram-based approach for SC classification. CCV methods are based on the principle of optimal feature engineering extraction and classification, and this is a crucial challenge that requires domain experts to resolve correctly. Further, due to the limited performance (Accuracy, Precision, Recall, Sensitivity, and Specificity) of the CCV-based method, the CAD system cannot be easily implemented in real-life scenarios.

In order to deal with these issues, different researchers used Deep Learning (DL)-based methods that showed promising results in several fields, such as disaster management [15,16], sentiment analysis [17], medical image analysis [18,19,20], energy management [21,22,23], forecasting [24,25], person-reidentification [26], segmentation [27], and specifically in the field of SC classification [28,29,30,31,32,33]. Compared to conventional machine learning-based methods [30,34], the performance of DL-based methods is much better. In addition, the information fusion method of the DL-based method has also proven to be of great importance in medical diagnostics [35,36]. Recently, Haug et al. [37] presented a DL-based method for the classification of SCs that is efficient. They used two pre-trained models called DenseNet and EfficientNet and optimized their features according to those models. As an experimental evaluation, they used the HAM10000 dataset and achieved an accuracy of 85.8%. A major objective of this work was to make it possible to apply it to low-cost devices such as smartphones. Jetson Nano. Carcagn et al. [38] proposed a CNN-based method for multiclass SC classification. DensNet CNN architecture was used, fine-tuning was done according to the problem, and an SVM classifier was employed for the final classification. In their experiments, they provided a dataset from HAM10000 and obtained a 90% accuracy rate. However, their method seemed to perform much better on balanced class datasets. In [39] was proposed an ensemble of DL-based models for multiclass SC classification. The researchers used five trained deep learning models such as MobileNetV2, Inception-ResNetV2, DenseNet201, Inception3, and GoogleNet, and tuned them according to the problem. In addition,, they used a plain classifier and a hierarchy of classifiers to classify the data. HAM10000 was used during the experiments, and they achieved an accuracy of 87.7%. DenseNet models have attained good results in experiments, and can be useful when balancing the datasets. A DL-based method for multiclass SC classification was presented by Mohammed et al. [40]. This study proposed a two-tiered framework to train models on all deeply connected layers and to resolve the issue of an imbalanced dataset. In the second step, they applied two pre-trained models, such as MobileNet and DenseNet121, for classification. As a result of the balanced training data, they achieved 92.7% accuracy over the HAM10000 dataset. The proposed model has the potential to be used in mobile applications. A method based on DL-based classification was presented by Chaturvedi et al. [41] for multiclass SC classification. As a first step, the models normalize the input images and resize them according to the DL models. A total of five different pre-trained models are then used to extract features and to classify them. The accuracy reached 92.83% using the balanced dataset HAM10000. One of the major contributions of this work is the integration of different DL models for better results evaluation by fusing information from different DL models. Next, [42] employed two DL models in order to classify the SCs, namely InveptionV3 and ResNet50. Using ISBI2018 and HAM10000 datasets, their method achieved an accuracy of 89.05% and 89.90%, respectively. Almaraz-Damian et al. [43] proposed a fusion framework for SC classification based on a dermoscopic image for the first stage. They combined well-known clinical features such as Asymmetry, Border, Color, and Diameter (ABCD) and handcrafted features to evaluate results more accurately. In the next stage, DL-based features were extracted and fused with the first stage. Classification was performed using the relevant vector machine and SVM classifier, which achieved 92.4% accuracy on the ISBI2018 dataset. Researchers in [44] developed a residual DL framework for SC classification and achieved 93% accuracy over the ISBI2018 dataset. Agrahari et al. [45] used the MobileNet model for efficient SC classification and performed experiments on the HAM10000 dataset, achieving 80.81, 91.25 and 96.26%, “top 1”, “top 2”, and “top 3” accuracy, respectively. In another approach [46], researchers used the data” augmentation technique to solve the data imbalance problem in the dataset. They used the pre-trained weight of several DL-based models, and Xception Net achieved promising results in the experiment. Nawaz et al. [47]., proposed a hybrid model for SC classification based on DL and fuzzy k-means clustering algorithms and achieved 95.6, 93.1 and 95.40% accuracy on the PH2, ISIC-2017 and ISIC-2016 datasets, respectively. Another hybrid model for SC classification was proposed by Sharma et al. [48] who fused the features of cascaded ensembling of CNN and a handcrafted features-based DL model and achieved state-of-the-art performance. There is no doubt that vision transformers play an important role in several vision-based challenging applications, such as fire detection [49,50], anomaly detection [51], and medical image classification [52,53]. It is well documented that, according to the recent literature, multiclass SC classification is not an easy task because of the large amount of similarity in the dermoscopic images. The second problem faced in the studies mentioned above is imbalanced datasets. Several researchers used the data augmentation method to deal with imbalanced datasets but, unfortunately, a low-class selection was used to deal with the problem, leading to overestimation of the likelihood of the dataset. This is not an optimal strategy because some of the images were overlooked during the training process. In this work, we propose an extensive evaluation of the MVT model to effectively classify SC images. In this work, the images under analysis were split into nine patches, then these patches transformed into a sequence by flattening and embedding. To keep positional information, position embedding was added to the patches. The acquired patch sequences were then fed to a number of Multi-Head Self-Attention (MHSA) layers to generate the final representation. In classification, the first sequence of the token is fed as an input to a classifier which classifies the given input images into their corresponding classes. The major contributions of this work are as follows:

- The major problem in the field of SC is the lack of publicly available datasets. Although there are some datasets available on the internet with a limited number of samples, these datasets are imbalanced, which negatively affects the performance of a model Thus, we used extensive data augmentation by augmenting the data with various parameters and different techniques to fill the data gap and make the system transformational and noise invariant.
- The current literature focuses on CNN-based models for SC classification, but these models reduce the dimensionality of the input data, which causes loss of meaningful information. To fill this gap we developed a vision transformer-based model with the potential of learning from a whole set of features, providing better accuracy.
- We evaluated the proposed model on a publicly available HAM10000 dataset and obtained higher performance in terms of F1-score, specificity, sensitivity, and accuracy when compared to state-of-the-art methods.

The rest of the work is arranged in the following manner. Section 2 discusses our method. Section 3 deals with the results and analysis of our experiment. Finally, in Section 4, we present the conclusions of our experiment.

## 2. The Proposed Method

**Figure 1:**
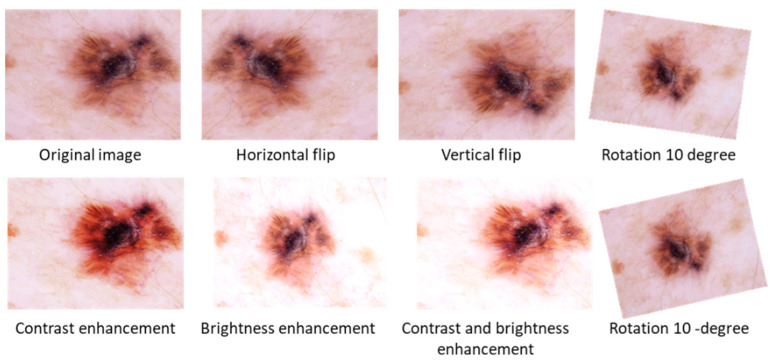
Visual effects of data augmentation steps.

**Figure 2:**
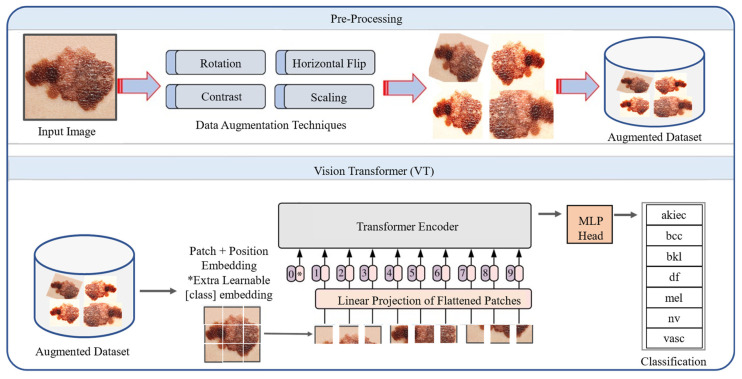
Proposed MVT-based SC classification framework.

In this section we describe data preprocessing steps and our proposed framework, as shown in Figure 1 and Figure 2. The proposed framework consists of two main phases, including data preprocessing as well as training the MVT model for the classification of SCs.

### 2.1. Data Preprocessing

Pre-processing of data is an important step in machine learning that enhances the quality of the data to be used. When training the CNN model on raw input data, for example, classification accuracy may decrease. Therefore, in the preprocessing stage, data augmentation is used to generate supplementary images from the existing images to vary their scale, position, orientation, contrast enhancement, and brightness adjustment, as shown in Figure 1.

***Brightness adjustment:*** Different lighting conditions can cause brightness variation in images and gamma correction transformation (1) with different values generates under-lit and overly lit images as demonstrated in Figure 1. Hence, the selected images can have such illumination-induced variations. By using brightness adjustment transformation we can improve the variations.

(1) g(x,y)=a×f(x,y)

***Contrast Enhancement:*** This transformation is used to adjust effects of contrast variations in images due to varying conditions of lights. The contrast stretching mechanism in Equation (2) is used to adjust contrast variation through different factors as shown in Figure 1.

(2) $$g\left(x,y\right)=\left\{\begin{array}{c} z1f\left(x,y\right)f\left(x,y\right)<r1\\ z2\left(f\left(x,y\right)-r1\right)+s1,r1\geq f\left(x,y\right)<r2\\ z3\left(f\left(x,y\right)-r2\right)+s2,f\left(x,y\right)\geq r2 \end{array}\right\}$$

where *f*(*x*,*y*) is the input and *g*(*x*,*y*) is the output pixel value, *s*1, *s*2, *r*1, and *r*2, are the user defined parameters for contrast adjustment, *z*1, *z*2 and *z*3 are the scale factors for the rotations in grayscale image and formulated as *s*1/*r*1, (*s*2 − *s*1)/(*r*2 − *r*1), (*L* − *s*2)/(*L* − *r*2), respectively, and L is the maximum range of gray level value.

***Geometric Transformations:*** This transformation includes translation, and rotation scaling was used on each image of the dataset to obtain new images as shown in Figure 1. For the CNN architecture, this step is very valuable to read the same object from different perspectives, which enhances the generalization capabilities of the model.

### 2.2. Our Proposed MVT Model for SC Classification

Transformer architecture was proposed by Vaswani et al. [54], which is an encoder-decoder module, and transforms a given sequence of elements into another sequence. The major theme behind the transformers is to enable parallel processing for the data. In this research, we explored vision-based Transformer architecture for SC classification as shown in Figure 2, where the MVT architecture receives an image as input data with a size of 72 by 72. First, the input image is converted into patches as in [55]. MVT supports a different number of patches based on the underline scenario. We split the input image into nine patches. To deal with 2D images, the image $X\in \;{\mathbb{R}}^{\left(H\times W\times C\right)}$ is reshaped as a sequence structure, similar to word embeddings, to the transformers input 2D patches $X_{p}\in \;{\mathbb{R}}^{N*\left(P^{2}.C\right)}$, where (H,W)  represents the resolution of the original image, and the resolution of image patches are represented by (P,P). $N=HW/P^{2}$ is the effective length of sequence for the transformer. The transformer treated these patches in the same way as tokens in natural language processing. The transformer uses a constant width in each layer and a trainable linear projection maps each vectorized path to the model dimension D, the output of which is referred to as patch embeddings. The MVT comprised an embedding layer, encoder layer, and classifier layer, which are described as follows:

***Embedding Layer:*** The transformer processes each patch as an individual token and maps to dimensions D with a learnable linear projection E. The embedded projections are fused with a learnable class token $U_{class}$, which is a key to completing the classification process. The positional embedding $E_{pos}$ is used to track and maintain the arrangement of each patch to identify the actual image. The patch encoded concatenation, with the token $Z_{0}$, is given in the following equation.

(3) $$Z_{0}=\left[U_{class};\;X_{p}^{1}E;\;X_{p}^{2}E;\ldots .;X_{p}^{N}E\right]+E_{pos}$$

***Encoder Layer:*** In this step, the transformer encoder is used to receive a series of embedded patches $Z_{0}$. The MVT creates L number of encoder blocks, then these blocks are divided into two subcomponents such as MHSA and the Multi-Layer Perceptron (MLP).

The MHSA block is the key element of the transformer encoder and contains self-attention and concatenation layers. The self-attention receives an input of *x* = *x*1, *x*2, … *Xn*, the transformer is responsible to makes an attention operation simultaneously on a set of queries  (Q) with all keys (K), and values (V), as formulated in Equation (4).

(4) $$Atten\;\left(Q,\;K,V\right)=softmax\;\left(\frac{QK^{T}}{\sqrt{D}}\right)V$$

Here *Atten* is a self-attention and $W^{Q}$, $W^{K}$, $W^{V}$ are weight matrices to be learned that attain the weights on the value. First, a dot product of Q across all K is calculated, then the square root of D is used to scale, and finally a SoftMax classifier is used. The transformer runs multi-head through scaled dot product attention many times in parallel with different weights. All these attention heads are fused to generate the final output, as formulated in Equation (5).

(5) $$\mathrm{MHSA}\;\left(Q,\;K,V\right)=conc\left(Atten1,\ldots \;Attenth\right)W^{O}$$

where MHSA is the combined attention heads and $W_{i}^{Q}$, $W_{i}^{K}$, $W_{i}^{V}$ and $W^{O}$ are the learning parameter matrices.

The encoder part is composed of identical layers, since *L* and has two key subcomponents: a multi-head self-attention block (MHSA), and a fully connected feed-forward dense block (MLP), as shown in the Equations (6) and (7). The blocks consist of two dense layers followed by GeLU activation, where a skip connection is used in the encoder and the output is preceded by layer normalization (*LN*).

(6) $${z’}_{l}=\mathrm{MHSA}\;\left(LN\left(Z_{l-1}\right)\right)+Z_{l-1},\;where\;l=1,\;2,\;3,\ldots ,L$$

(7) $$z_{l}=\mathrm{MLP}\;\left(LN\left({z’}_{l}\right)\right)+{z’}_{l},\;where\;l=1,\;2,\;3,\ldots ,L\;$$

***Classification layer:*** From the sequence, the first item $Z_{l}^{0}$ is taken and fed to an external head classifier to anticipate the encoder’s final layer for classification, which classifies the class label into two corresponding class labels, such as fire or non-fire, and is formulated as below.

(8) $$y=\mathrm{Layer}\;\mathrm{Normalization}\;\left(Z_{l}^{0}\right)$$

Here, *y* is the output of the model and $Z_{l}^{0}$ the first item taken for the decision.

In this work, we utilized and fine-tuned the MVT large model hyperparameter of the proposed model based on several experiments with the following selections: layer (24), hidden size *D* (1024), MLP size (4096), Heads 16, and Parameters 86 million.

In the Algorithm 1, we provide the training and testing of the steps of the proposed MVT model.
**Algorithm 1 Training and testing of the proposed MVT model**Input: Dataset of SC images Dataset split: Training, Validation and TestingOutput: predicted labelsTraining:   1.Model parameters
Image size: (72,72,3)P: 9Mini-batch size: 32N; number of samplesLearning rate: 0.0001Optimizer: AdamW
   2.Set the number of mini-batches as: $N_{b}=\frac{N}{b}$
   3.For iteration = 1: number of epochs       For batch = 1 number of mini-batches
Image augmentationThe obtained training set is fed to the MVT encoder’s class branchThe augmented images batch is fed to the MVT encoder of the classification branchThe classification token is fed the token classifierCalculated the loss functionLoss backpropagationUpdating the model parameters
Model testing:   1.Feed the input images to the model
   2.Calculate the prediction label using output label Y

## 3. Experimental Setup and Results

**Figure 3:**
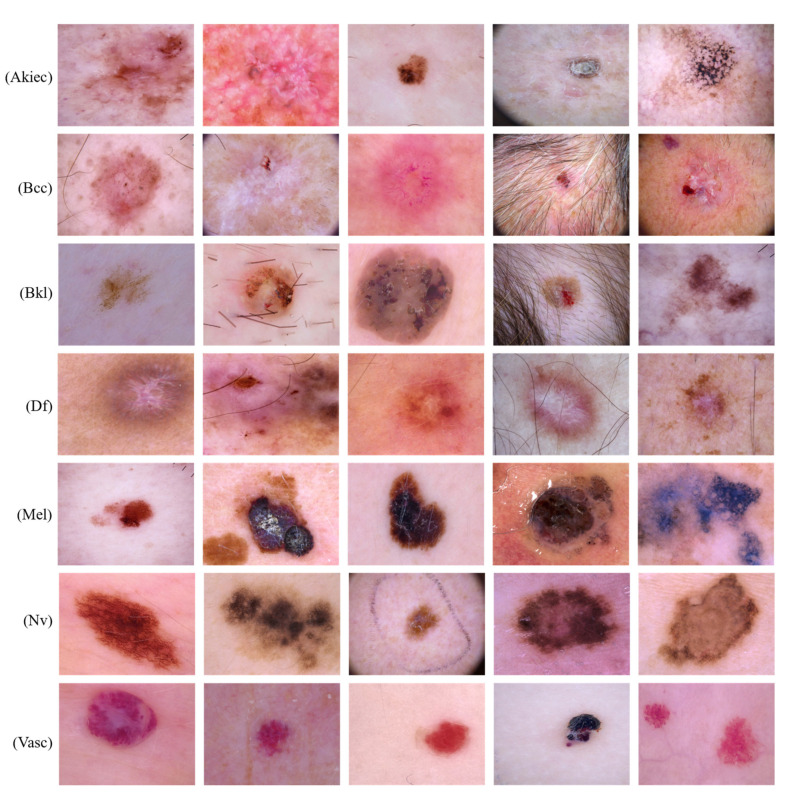
Sample images from the HAM10000 dataset.

In this section, we provide a detailed explanation of the implementation and classification of the proposed method. The dataset used for the experimental evaluation was HAM10000 [34]. Several classes in the dataset are highly imbalanced, such as Dermatofibroma (Df), Melanoma (Mel), Melanocytic nevi (Nevi), Actinic keratoses (Akiec), Vascular lesions (Vasc), Basal cell carcinoma (Bcc), and Benign keratosis-like lesions (Bkl). During the analysis of the dataset, a total of 10,015 images were obtained. To improve the generalization of the model, we provided some preprocessing steps to increase visibility and the number of training examples. Moreover, we also addressed the issue of data imbalance through preprocessing steps. We did not make any preprocessing step for the Bkl, Nv, and Mel classes in the dataset, which can be seen in Table 1, because these classes have a high number of images. Table 1 shows the number of images in the original dataset and the number of images in the preprocesses dataset. As a result, 70% of the data were used for training, 20% for validation, and 10% for testing. Figure 3 shows sample images from the HAM10000 dataset.

It is worth mentioning that all experiments were carried out with Python 3.6, TensorFlow with Keras front ends on a Core i5-7200u Central Processing Unit (CPU) (2.7 GHz) with a main memory of 8 GB and a GeForce (2060 gtx) Graphic Processing Unit (GPU) of 6 GB. To evaluate the performance, we employed different evaluation matrices, namely precision, recall, F1-measure, and accuracy, which were obtained from the confusion matrix as discussed in [56,57,58] and formulated in the following equation, and were previously employed by several researchers for different vision-based classification problem.

(9) $$Precision=\left(\frac{True\;positive}{True\;positive+False\;positive}\right)$$

(10) $$Recall\;or\;Sensitivity=\left(\frac{True\;positive}{True\;positive+False\;negative}\right)$$

(11) $$F1-measure=2*\left(\frac{Precision*Recall}{Precision+Recall\;}\right)\;$$

(12) $$Acc=\left(\frac{True\;positive+True\;negative}{True\;positive+True\;negative+False\;negative+False\;positive}\right)\;$$

### 3.1. Results Evaluation with and without Preprocessing

**Figure 4:**
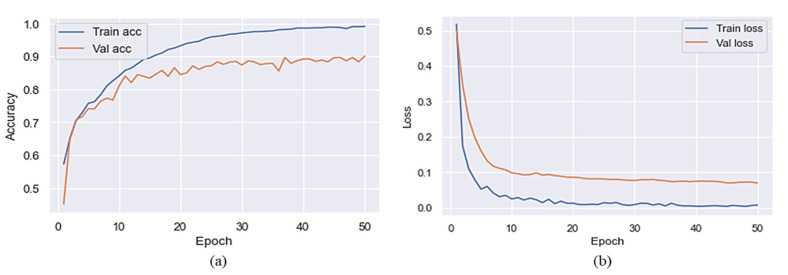
Training/validation accuracy and loss of the proposed model without the preprocessing (**a**) accuracy and (**b**) loss.

This section explains how we used a pre-trained MVT model for SC classification. We used 50 epochs to train the model on the target dataset before and after preprocessing. Based on the results reported in Table 2 and Table 3, it is clear that MVT is effective with and without data preprocessing. The accuracy of the proposed model is presented in Figure 4 without preprocessing steps to represent the training and validation accuracy. Figure 4 shows the *x*-axis representing a number of epochs and the *y*-axis representing accuracy and loss over time. As shown in this Figure, the blue color indicates training accuracy and loss, whereas the orange color represents accuracy and loss during validation. The validation accuracy was higher than the accuracy of training at the initial stage of a training process after a few epochs had passed. It can be observed that the training accuracy reached 80% after five epochs, but a sudden reduction can be seen in the validation accuracy, which was 77% after five epochs. Training and validation accuracy of the proposed model, without the use of preprocessing, reached 98% and 90%, respectively. The validation loss throughout the experiment was higher than the training loss when considering the loss of training and validation. On the final epoch, the training and validation loss reached 0.02 and 0.1, respectively. Due to this, the proposed model introduced overfitting after 20 iterations.

Table 2 shows the confusion matrix for the proposed model without using the preprocessing step, and shows the misprediction value and the exact value for each class. Based on the data n Table 2, it can be seen that the Mel class had the lowest accuracy and the Df and Vasc classes had the highest accuracy. As a result, the overall test accuracy was achieved at 90.28%.

**Figure 5:**
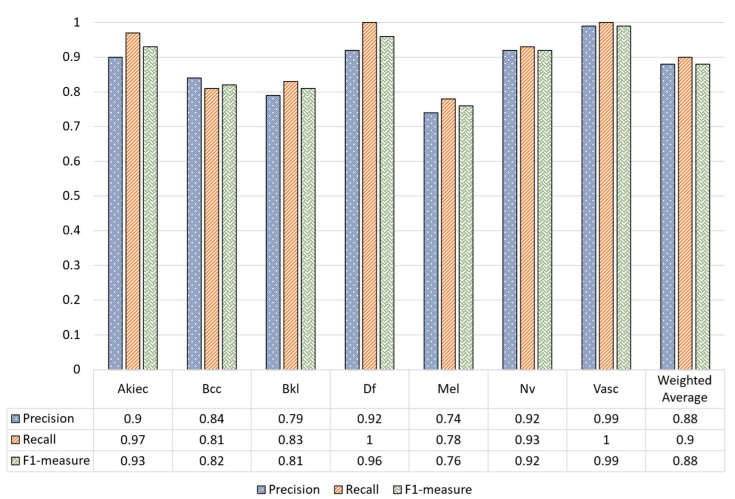
Classification report of our model used without the preprocessing stage.

The classification report of our model using the test set of the unaugmented dataset is given in Figure 5, where the recall, precision, and F1-measure of each class are given. In Figure 5, it can be observed that the lowest recall, precision, and F1-measure was achieved by the Mel class whereas the Vasc and Df classes achieved the highest performance in terms of all evaluation metrics due to a large number of training examples.

**Figure 6:**
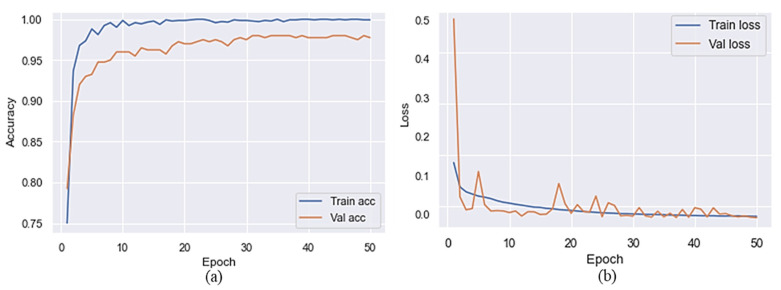
Training/validation accuracy and loss of the proposed model with the preprocessing (**a**) accuracy and (**b**) loss.

Figure 6 shows the training and validation accuracy of the proposed model with preprocessing steps. At the end of the first epoch, the accuracy of the training algorithm was 75%, while the accuracy of the validation algorithm was 79%. When the epoch increased, a significant improvement was found in training and validation accuracy. During the final epoch, the training accuracy reached 99% and the validation accuracy reached 97%. In addition, with regards to the loss, it is can be seen in Figure 6 that the training and validation losses were significantly reduced. By the end of the training and validation epochs, the training and validation loss had reached 0.01.

**Figure 7:**
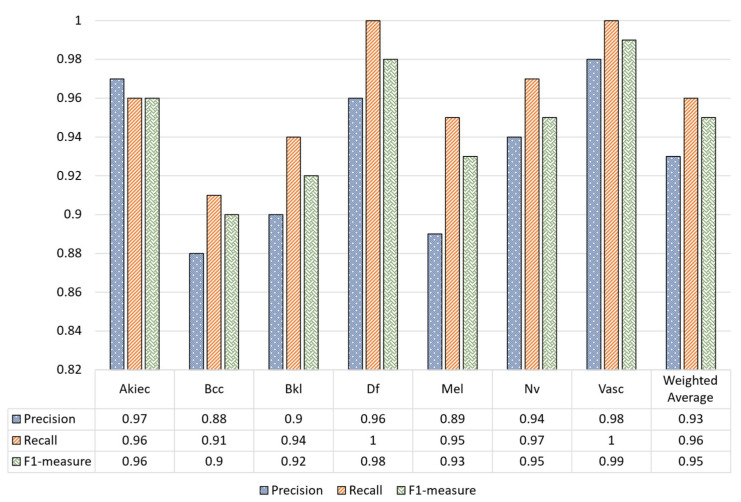
Classification report of our model using the preprocessing stage.

The classification report in Figure 7 shows that the proposed MVT model over the augmented dataset achieved higher performance for precision, recall, and F1-measure compared to the unaugmented dataset.

Table 3 shows the outcomes of data preprocessing steps based on MVT. It is noteworthy that the performance of the SC classification significantly improved after MVT preprocessed the data. The confusion matrix of the proposed model using the preprocessing stage is given in Table 3. The matrix shows the correct and misprediction values of each class. As can be seen from Table 3, the lowest accuracy was achieved by the Bcc class, while the highest accuracy was reached by the Df and Vasc classes, respectively. The overall test accuracy was 96.14%. The classification report of the proposed model is given in Table 4, where the precision, recall, and F1 measure of each class can be observed.

### 3.2. Comparison of the Proposed Method with State-of-the-Art Methods

In this section, we discuss the comparison between the proposed model and state-of-the-art methods. As can be seen in Table 4, the proposed model achieved the highest performance in terms of precision, recall, F1-measure, and accuracy when compared to state-of-the-art methods. According to the proposed model, it achieves 96.14% accuracy, a 97.0% F1-measure, 96.50% recall or sensitivity, and 96.0% precision.

**Figure 8:**
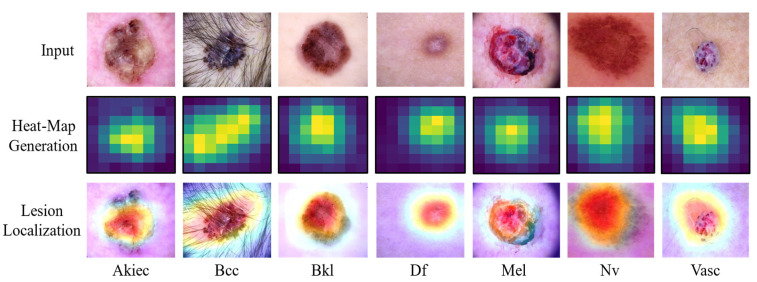
Samples of a heat-map for each class.

We conclude that our model outperforms state-of-the-art methods by 3.78, 12.3, 8.97, and 0.34% respectively. Additionally, we used the Grad-CAM visualization mechanism to generate a heat-map of our proposed model which was applied to the target dataset. A heat-map is used to precisely locate an SC lesion by identifying its precise location on the heat map. Figure 8 shows samples of a heat-map for each class. It is clear from these visual results that the proposed model is robust and effective in the area of SC classification.

## 4. Conclusions and Future Work

Machine learning algorithms have the potential to contribute greatly to augmenting the capabilities of medical experts in detecting early signs of skin cancer. This paper discusses a framework for skin cancer classification based on dermoscopic images using an MVT-based framework. The proposed method was evaluated on the HAM10000 dataset and achieved state-of-the-art performance. Compared to state-of-the-art methods, our method outperformed these in terms of precision, recall, F1measure, and accuracy by 3.78, 12.3, 8.97, and 0.34%, respectively. Our method improved when the number of images in all classes was increased to overcome the problem of data imbalance. The proposed MVT has a large number of training parameters and model size, and also requires a huge amount of training data that is not applicable to running on edge devices. In the future, we will employ model pruning and quantization techniques to overcome these challenges. Furthermore, we will employ enhanced level data augmentation techniques to overcome the problem of inequities in the data and thus improve the performance of SC classification.

## Figures and Tables

**Table 1:** Number of images for each class before and after the preprocessing stage.

No.	Class	Number of Images	Number of Images
		Before Preprocessing	After Preprocessing
1	Akiec	327	1099
2	Bcc	541	1099
3	Bkl	1099	1099
4	Df	155	1099
5	Nv	6705	6705
6	Mel	1113	1113
7	Vasc	142	1099

**Table 2:** Confusion matrix of the proposed model without the preprocessing stage.

Class	Akiec	Bcc	Bkl	Df	Mel	Nv	Vasc	Class-Wise Accuracy
Akiec	0.97	0.02	0.0	0.0	0.0	0.01	0.0	97.00%
Bcc	0.09	0.81	0.07	0.0	0.02	0.00	0.01	81.00%
Bkl	0.02	0.02	0.83	0.0	0.05	0.08	0.0	83.00%
Df	0.0	0.0	0.0	1.0	0.0	0.0	0.0	100%
Mel	0.02	0.0	0.08	0.0	0.78	0.12	0.0	78.00%
Nv	0.0	0.0	0.02	0.0	0.04	0.93	0.01	93.00%
Vasc	0.0	0.0	0.0	0.0	0.0	0.0	1.0	100%

**Table 3:** Confusion matrix of the proposed model with the preprocessing stage.

Class	Akiec	Bcc	Bkl	Df	Mel	Nv	Vasc	Class-Wise Accuracy
Akiec	0.96	0.01	0.0	0.0	0.0	0.02	0.1	96.00%
Bcc	0.01	0.91	0.04	0.01	0.02	0.01	0.0	91.00%
Bkl	0.02	0.02	0.94	0.0	0.01	0.01	0.0	94.00%
Df	0.0	0.0	0.0	1.0	0.0	0.0	0.0	100%
Mel	0.0	0.0	0.03	0.0	0.95	0.02	0.0	95.00%
Nv	0.0	0.0	0.0	0.0	0.02	0.97	0.01	97.00%
Vasc	0.0	0.0	0.0	0.0	0.0	0.0	1.0	100%

**Table 4:** Comparison of the proposed method with state-of-the-art methods.

Reference	Precision	Recall/ Sensitivity	F1-Measure	Accuracy
Attique et al. [59]	92.22	84.20	88.03	95.80
Gupta et al. [60]	89.00	83.00	83.00	83.10
Chaturvedi et al. [41]	88.00	88.00	88.00	93.20
Huang et al. [37]	75.18	--	--	85.80
Carcagni et al. [38]	88.00	76.00	82.00	90.00
Shahin et al. [42]	86.20	79.60	82.90	89.90
Jain et al. [46]	88.76	89.57	89.02	90.48
The proposed method	96.00	96.50	97.00	96.14

## Data Availability

Not applicable.
